# Pathogenic Microbes Increase Plant Dependence on Arbuscular Mycorrhizal Fungi: A Meta-Analysis

**DOI:** 10.3389/fpls.2021.707118

**Published:** 2021-10-04

**Authors:** Mingsen Qin, Jean-Pascal Miranda, Yun Tang, Wangrong Wei, Yongjun Liu, Huyuan Feng

**Affiliations:** ^1^Key Laboratory of Southwest China Wildlife Resources Conservation, Ministry of Education, China West Normal University, Nanchong, China; ^2^Department of Biology, University of York, York, United Kingdom; ^3^Key Laboratory of Cell Activities and Stress Adaptations, School of Life Sciences, Lanzhou University, Lanzhou, China

**Keywords:** arbuscular mycorrhizal fungi, pathogenic microbes, meta-analysis, effect size, plant growth

## Abstract

Numerous studies have confirmed that arbuscular mycorrhizal fungi (AMF) can promote plant nitrogen and phosphorus absorption, and prime systemic plant defense to plant pathogenic microbes. Despite that, the information on the interaction between AMF and plant pathogenic microbes is limited, especially the influence of plant pathogenic microbes on the effect of AMF promoting plant growth. In this study, 650 independent paired-wise observations from 136 published papers were collected and used to calculate the different effect of AMF with plant pathogenic microbes (DAPP) in promoting plant growth through meta-analysis. The results showed that AMF had a higher effect size on plant growth with pathogenic microbes comparing to without pathogenic microbes, including the significant effects in shoot and total fresh biomass, and shoot, root, and total dry biomass. The results of the selection models revealed that the most important factor determining the DAPP on plant dry biomass was the harm level of plant pathogenic microbes on the plant dry biomass, which was negatively correlated. Furthermore, the change of AMF root length colonization (RLC) was the sub-important factor, which was positively correlated with the DAPP. Taken together, these results have implications for understanding the potential and application of AMF in agroecosystems.

## Introduction

Feeding an increasing global human population while maintaining the sustainability of the farmland is the most important challenge in the twenty-first century (Godfray et al., 2010; Tilman et al., 2011). The primary limiting factor in this challenge is the poor availability of soil nitrogen (N) and phosphorus (P) needed to increase crops yields (Bennett et al., 2013). Consequently, arbuscular mycorrhizal fungi (AMF) have a great potation for more efficient agriculture (Rodriguez and Sanders, 2014), because AMF are the one of the key mechanisms of enhancing the acquisition of N and P by crops (Smith and Read, 2008; Hodge and Fitter, 2010; Zhang et al., 2019) and also can improve mineral acquisition in plants (Clark and Zeto, 2000; Lehmann and Rillig, 2015). AMF form mutualistic associations with the roots of over 80% of land plant species (Smith and Read, 2008) and provide N and P to host plant in return for lipids and/or sugars (Bago et al., 2000; Jiang et al., 2017). However, plant pathogenic microbes are wildly distributed in agriculture system and are the cause of least to 15% crop yield losses globally (Savary et al., 2019). In comparison with the function of absorption on N and P, there are few researches focusing on the effect of AMF on plant pathogenic microbes (Dugassa et al., 1996; Veresoglou and Rillig, 2012), especially on the interaction between them and how plant pathogenic microbes may influence the plant growth promotion function of AMF. Therefore, systematic research on the interaction between AMF and pathogenic microbes, particularly the influence of plant pathogenic microbes on function of AMF, can enrich our poor understanding of the application of AMF in pathogen protection.

Normally, the presence of AMF can lessen the harm of plant pathogenic microbes on plant growth (Newsham et al., 1995; Dugassa et al., 1996; Veresoglou and Rillig, 2012). Whether this effect is caused by the role of AMF in promotion plant growth, or by a specific microbe–microbe function is still unclear. Pozo et al. (2009) and Campos-Soriano et al. (2012) have found that AMF-associated plants will employ a more efficient defense response against plant pathogenic microbes. This mechanism indicates that AMF may play a direct role in the defense reaction to the plant pathogenic microbes, rather than solely aiding in promoting plant growth. Thus, we hypothesize that AMF may play a more efficient role in promoting plant growth in environments with plant pathogenic microbes in comparison with without pathogens. Furthermore, we also hypothesize that an increased AMF effect due to plant pathogenic microbes would be correlated with the harm level of the pathogens and the effect size of AMF on plant growth, because a greater harm caused by plant pathogenic microbes on plant growth may stimulate a higher defense reaction from plant colonized by AMF through producing more defensive compounds (Gianinazzi-Pearson et al., 1996; Pozo et al., 2009; Campos-Soriano et al., 2012). In the meanwhile, logically the difference of the effect level of AMF on plant growth with pathogenic microbes may be also determined by the effect size of AMF expressed without pathogenic microbes. Therefore, it is necessary to investigate the key factors of this potential interaction.

For both AMF and plant pathogen, their influences on plant growth were normally determined by the abiotic and biotic factors, for example, nutrient condition, the species of host plant, species of AMF, and species of pathogenic microbes (Berg and Koskella, 2018; Jiang et al., 2018; Mommer et al., 2018; Qin et al., 2020). But if and how these factors further influence the interaction between AMF and plant pathogenic microbes on the plant growth is also unknown. In order to reveal a more quantitative understanding of the influence of plant pathogenic microbes on the function of AMF on plant growth, we conducted a global meta-analysis on published articles reporting the influence of AMF on plant biomass with and without plant pathogenic microbes. Furthermore, all the influences on the performance of AMF with plant pathogenic microbes in influencing plant growth including both abiotic and biotic factors were investigated. To do so, a database of the basic abiotic and biotic factors, changes of AMF root length colonization (RLC), and influence of AMF and plant pathogenic microbes on plant growth was constructed from collected data. Using these database, we aimed to address the following two questions: (1) What is the function difference of AMF on plant growth between with plant pathogenic microbes and without? (2) What is the main factor determining this function difference?

## Materials and Methods

### Data Collection

We searched for articles on the Web of Science database and China National Knowledge infrastructure database (CNKI)^1^ during July 2020 and updated on July 2021 to collect any published articles. The search terms were: “[plant AND (pathogen OR disease) AND AMF OR (AM fung*) OR arbuscular OR mycorrhiza* OR Glomeromycota].” To increase the data coverage, we also searched these terms on the Google Scholar during July 2021. The mean values of shoot, root, and total dry/fresh biomass and the corresponding SD and sample size (N) for AMF-inoculated and non-inoculated plants under pathogenic and non-pathogenic conditions were extracted from the target articles. Retrieved articles that fulfilled the following criteria were selected: (a) any target data about fresh or dry biomass of plant shoot, root, or total were reported; (b) plants from the AMF non-inoculation treatment had at a maximum of 2% AMF RLC (Lehmann and Rillig, 2015); (c) each potted experiment was conducted with only one plant species to avoid interspecies competition or complementary effects between different species; and (d) the experimental duration was less than 1year to avoid the influence of aboveground plant parts dying. Any additional biotic or abiotic treatment beyond AMF and pathogenic treatments was excluded to avoid any possible interactions with AMF or plant pathogenic microbes. Thus, only pot experiments were used for this research and 138 publications met these selection criteria (Supplementary Figure S1). Data showing in graphs were extracted using the GetData Graph Digitizer 2.24.^2^ For articles that reported only SE, the SD was calculated as follows: SD=SE * sqrt (N). If neither SD nor SE was reported, the SD was calculated as 10% of the mean value (Brown et al., 2014; Zhou et al., 2017). To increase our data coverage, unreported total biomass was combined by the shoot and root biomass data, and the SD for the total biomass was conservatively calculated according to Taylor series expansion as follows: SD_(total biomass)_=SD_(shoot biomass)_+SD_(root biomass)_, where shoot and root biomass were presumed to be two normally distributed variables (Lee and Forthofer, 2006).

### Moderators

We collected information on 15 moderators that we hypothesized could influence the difference of AMF effect on plant biomass under pathogenic and no-pathogenic conditions (DAPP). We collected the soil pH, soil available phosphorus (P), and soil sandy content. We grouped the soil pH into: acidic <6.6, neutral: 6.6–7.3, and alkaline >7.3, and soil sandy content into sandy (sandy soil ≥50% of total soil) and not sandy (sandy soil <50% of total soil) according to USDA criteria.^3^ We collected the duration of inoculated pathogenic treatment as the experimental duration, because this duration was the period that AMF and pathogenic microbes interacted. The host plant was grouped into annual herbaceous, perennial herbaceous, and woody according to the life type and also grouped into nitrogen fixer and no-nitrogen fixer according to the function of nitrogen fixing. As root systems are also reported to influence the plant response to AMF, we grouped the plants into taproot and fibrous root system by the method of Yang et al. (2015). AMF inoculation was grouped into single (single AMF species) and mixed (over one AMF species) according to the AMF species number (Lehmann et al., 2014) and grouped into different family according to the newest classification system.^4^ After that, AMF species was also grouped into rhizophilic, edaphophilic and ancestral guilds, which was mainly according to the biomass allocation strategies of AMF (Weber et al., 2019). The pathogenic type was collected and grouped into fungi, oomycetes, bacteria, virus, and any possible combination of these pathogenic types, and the infection area was grouped into shoot and root for each pathogenic microbe. We collected the information on the RLC under AMF inoculation and AMF+pathogenic condition (inoculated with both AMF and plant pathogenic microbes) and mean plant biomass under AMF and plant pathogenic inoculation, and control treatment and then defined the ∆RLC=ln[RLC_(AMF+pathogen)_/RLC_(AMF)_], the log response ratio (*RR*) of AMF=ln[biomass_(AMF)_/biomass_(control)_], and *RR* of pathogen=ln[biomass_(pathogen)_/biomass_(control)_]. These changes of RLC or biomass were used to test their relationship with DAPP. To meet the normality, the available P and experimental duration was square-root-transformed for the following analysis.

### Meta-Analysis

We used the log response ratio (*RR*) and its corresponding variance (*Var*; Hedges et al., 1999) to measure the influences of AMF and plant pathogenic microbe on the plant biomass. The *RR*=ln(*X*_t_) - ln(*X*_c_), where *X*_t_ is the mean plant biomass (shoot, root, and total, respectively) inoculated with AMF or plant pathogenic microbe, and *X*_c_ is the mean plant biomass in control treatment. The *Var* of each *RR* was calculated as: $Var\left(RR\right)=\frac{S_{t}^{2}}{N_{t}X_{t}^{2}}+\frac{S_{c}^{2}}{N_{c}X_{c}^{2}}$ , where $N_{t}$ is the sample size of the AMF or plant pathogenic microbe treatment and $N_{c}$ is the sample size of the control treatment, and $S_{t}^{2}$ is the SD of the AMF or plant pathogenic microbe treatment, and $S_{c}^{2}$ is the SD of the control treatment (Gurevitch et al., 2001). To calculate the difference of the effect of AMF on the plant biomass between pathogenic and no-pathogenic conditions (DAPP), we also use the paired-wise log-response ratio: DAPP=[ln(*X_P, A_*)-ln(*X_P_*)]-[ln(*X_A_*)-ln(*X*_C_)], where *X_P,A_*, *X_A_*, *X_P_*, *X*_C_ represent the mean value of plant biomass under the treatment of pathogen+AMF, AMF, pathogenic, and control, respectively; the *Var*(DAPP)= $\frac{S_{P,A}^{2}}{N_{P,A}X_{P,A}^{2}}+\frac{S_{A}^{2}}{N_{A}X_{A}^{2}}+\frac{S_{P}^{2}}{N_{P}X_{P}^{2}}+\frac{S_{c}^{2}}{N_{c}X_{c}^{2}}$ , the *S* and N represent the corresponding SD and sample size, respectively (Crawford et al., 2019). For DAPP, a positive value revealed that the inoculation of AMF had a higher effect on plant biomass with plant pathogenic microbes comparing to the no-pathogenic condition.

All analyses were performed with R-4.1.0 (R Core Team, Vienna, Austria). The mean *RR* and DAPP and their corresponding 95% CI were calculated using the *rma.mv* function from *metafor* package with a REML method, in which the articles and observation identity were considered as the random factors (Viechtbauer, 2010). If the 95% CI values did not overlap with zero, the mean *RR* or DAPP was considered significant (*p*<0.05). To calculate the importance of different moderator in impacting the DAPP, *rma.glmulti* function in *metafor*, using *glmulti* 1.0.8 (Calcagno and de Mazancourt, 2010), was used to conduct model selection based on the AICc. The full model for each dataset included DAPP as response variable; the moderators as fixed effects; the articles and observation identity as the random factors; and *Var*(DAPP) as the variance. During the model selection, models were ranked according to AICc and an average of the top 128 models for each dataset was calculated with *glmulti*. The estimates and 95% CI for each moderator were weighted based on the AICc weights across all the models. Moderators that appeared in the top 128 models were assigned an importance value, according to the sum of the weights for the models in which the variable appears. A moderator was classified as significantly contributing to variation in DAPP if it had an importance value>0.8 (Terrer et al., 2016; Crawford et al., 2019). While the variables of soil pH and available P are important for affecting the functions of AMF, they were unfortunately only reported in less than half observations in our dataset. Thus, these two moderators were not set into the model selection analysis. The number of observations on fresh biomass was less than the number needed for the model selection with our test moderators (Viechtbauer, 2010); thus, the model selection was not conducted on the fresh biomass-related observations. To test for the significant differences between levels of moderators or the linear relationship with moderators, we used the *rma.mv* function to analyze the effect model for each moderator with an importance value>0.8 from the selection model.

## Results and Discussion

**Figure 1:**
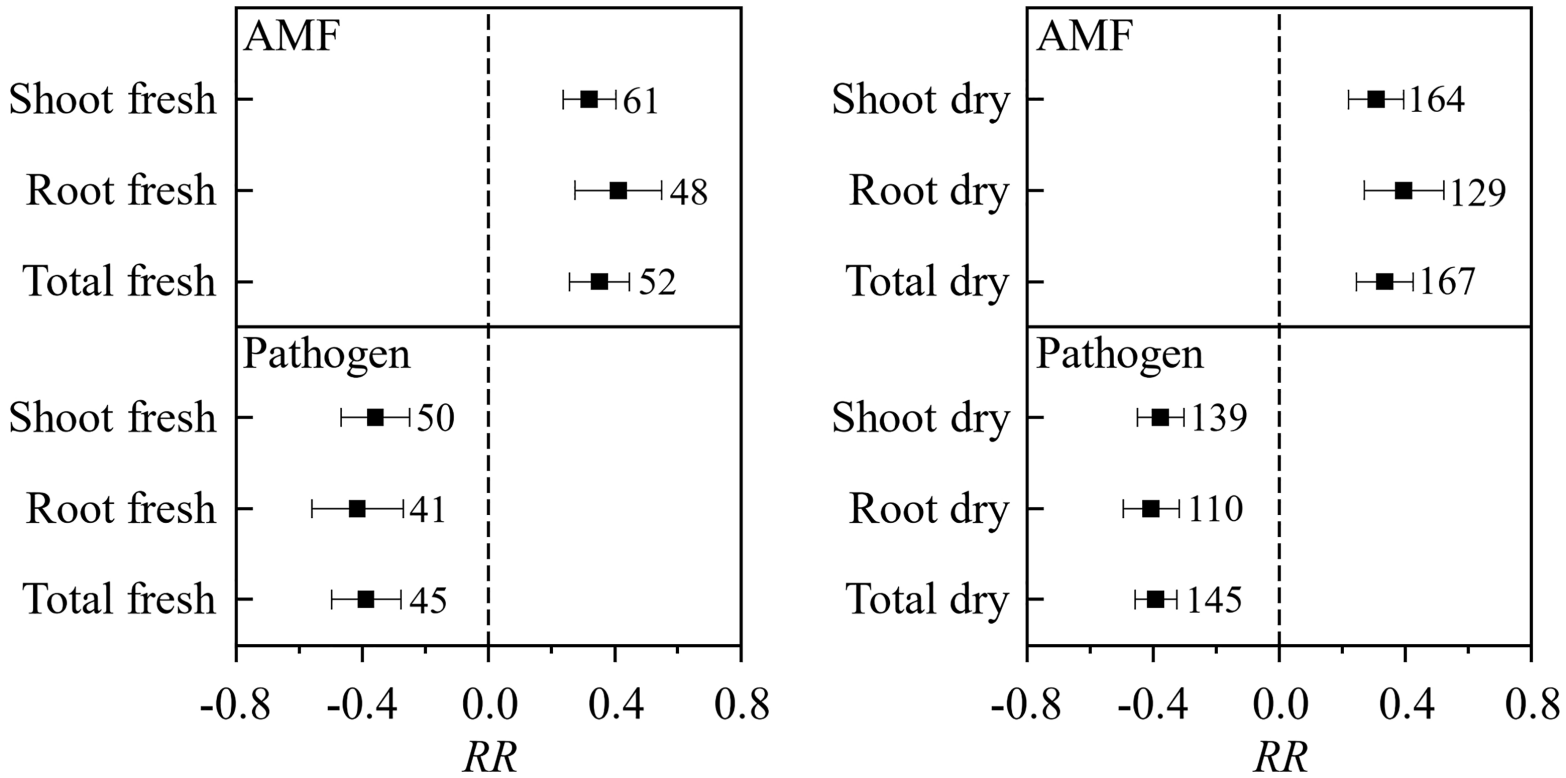
The effect of AMF and pathogenic microbes on plant fresh and dry biomass. Values near the error bar were numbers of observations included in the analysis.

**Figure 2:**
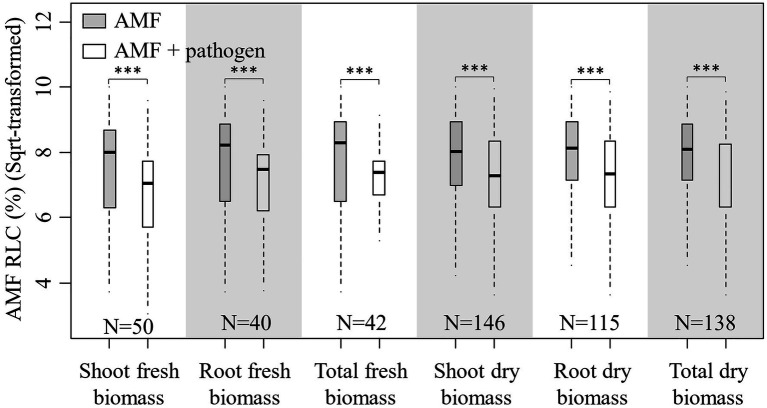
Boxplot for root length colonization (RLC; sqrt-transformed) of AMF inoculation and AMF+pathogen inoculation treatments for each sub-database. Paired *T* test is used to calculate the significant difference: ^***^p<0.001. N is the number of observations used for paired *T* test.

In total, 138 published papers met our criteria, and 650 independent paired-wise observations were included in this study (Supplementary Data File S1). The result on the effect of AMF and plant pathogenic microbe on plant biomass showed that the inoculation of AMF significantly increased the plant fresh and dry biomass, and plant pathogenic microbe decreased the plant fresh and dry biomass (Figure 1). These results fitted the previous results and confirmed that AMF had positive effect and plant pathogenic microbes had negative effect on plant growth (Mendes et al., 2013; Treseder, 2013; Zhang et al., 2019). We found that the RLC of AMF was reduced by the pathogenic microbes from the paired *T* test for combination of fresh and dry biomass for shoot, root, and total (Figure 2). This result revealed that host plant reduced the carbon offering to the AMF when colonized additionally with plant pathogenic microbes (Dar and Reshi, 2017; Singh and Giri, 2017).

**Figure 3:**
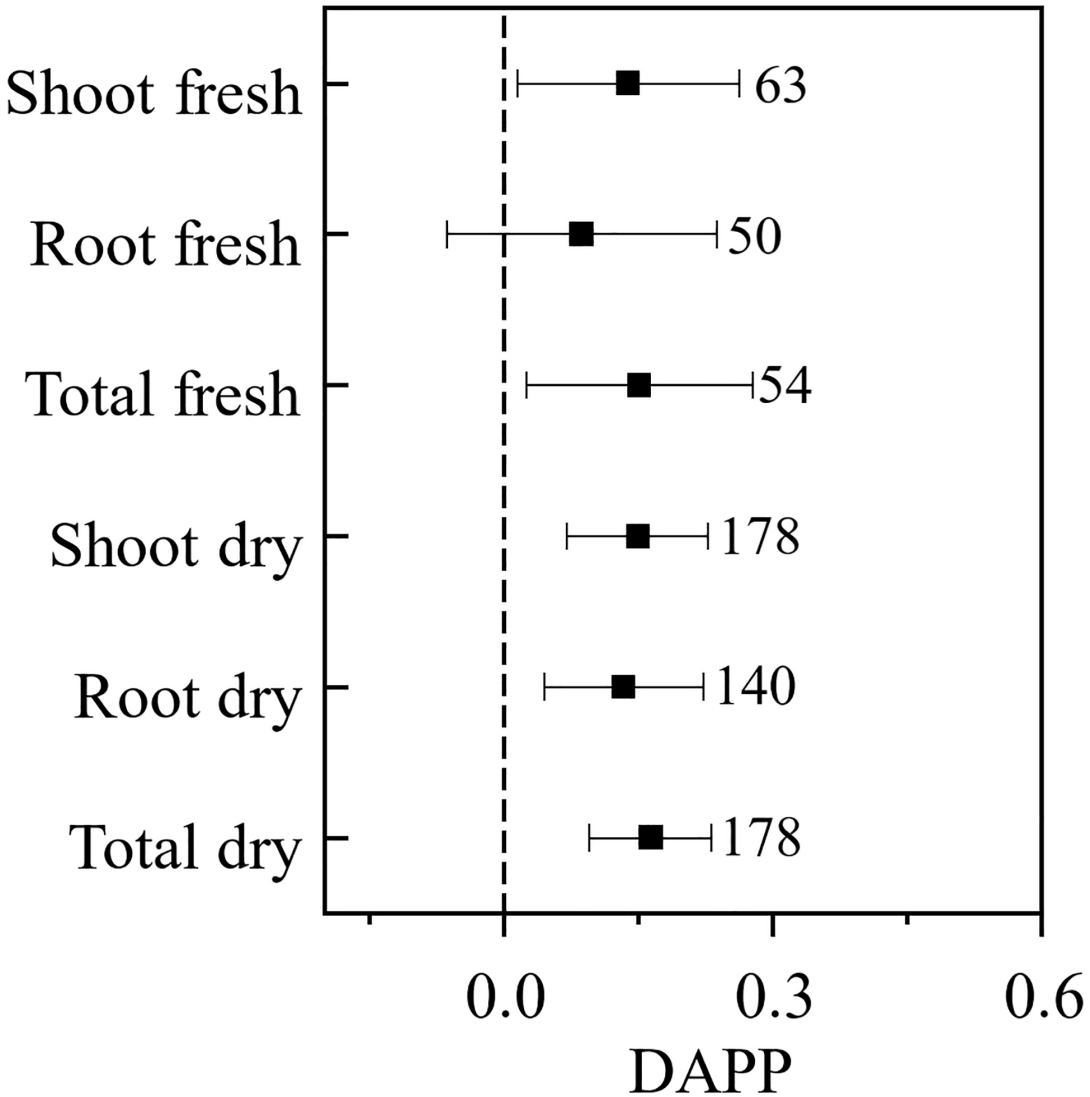
The difference of the effect size of AMF on the plant biomass under plant pathogenic microbes and no-pathogenic condition (DAPP). Values near the error bar were numbers of observations included in the analysis.

Our paired-wise comparison revealed that under the plant pathogenic condition AMF had higher effects on plant biomass, which was significant for the plant shoot (DAPP=0.138, CI: 0.015–0.262) and total (DAPP=0.151, CI: 0.025–0.277) fresh biomass, and shoot (DAPP=0.149, CI: 0.071–0.228), root (DAPP=0.133, CI: 0.044–0.223), and total (DAPP=0.163, CI: 0.096–0.231) dry biomass, and was nearly significant for root fresh biomass (DAPP=0.087, CI: −0.064–0.238; Figure 3). Taken together these results revealed that plant pathogenic microbes promoted the effect of AMF on plant growth, except for root fresh biomass. The effect size of AMF on plant growth is also regarded as the plant dependence on AMF; in another word, our results revealed that plant pathogenic microbes enhance the dependence of host plant on AMF. If only considering the influence of AMF on nutritional benefits for the host plant, the effect of AMF on a plant with pathogenic microbes should logically not be higher than that without pathogenic microbes. A likely explanation to our results is that AMF not only promote plant growth when plant pathogens are present, but also help to protect the plant from those pathogenic microbes (Saldajeno et al., 2008; Sikes, 2010) and compete for the same fatty acid resource with pathogenic microbes (Jiang et al., 2017), which presents an indirect mechanism to increase the effect of AMF on plant growth by reducing the harm of plant pathogenic microbes.

**Figure 4:**
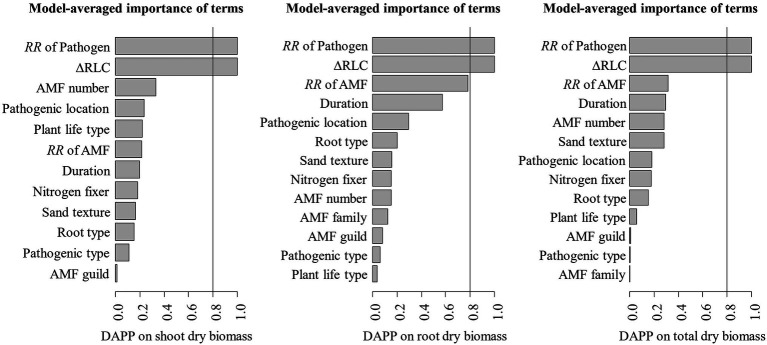
Variable importance of moderators for the effect size difference of AMF on the plant dry biomass under plant pathogenic microbes and no-pathogenic conditions (DAPP). The importance values are the sum of the weights for the models in which the variable appears. The averaged models included the top 128 candidate models. Moderators with an importance of 0.8 or greater are considered for the significance tests.

**Figure 5:**
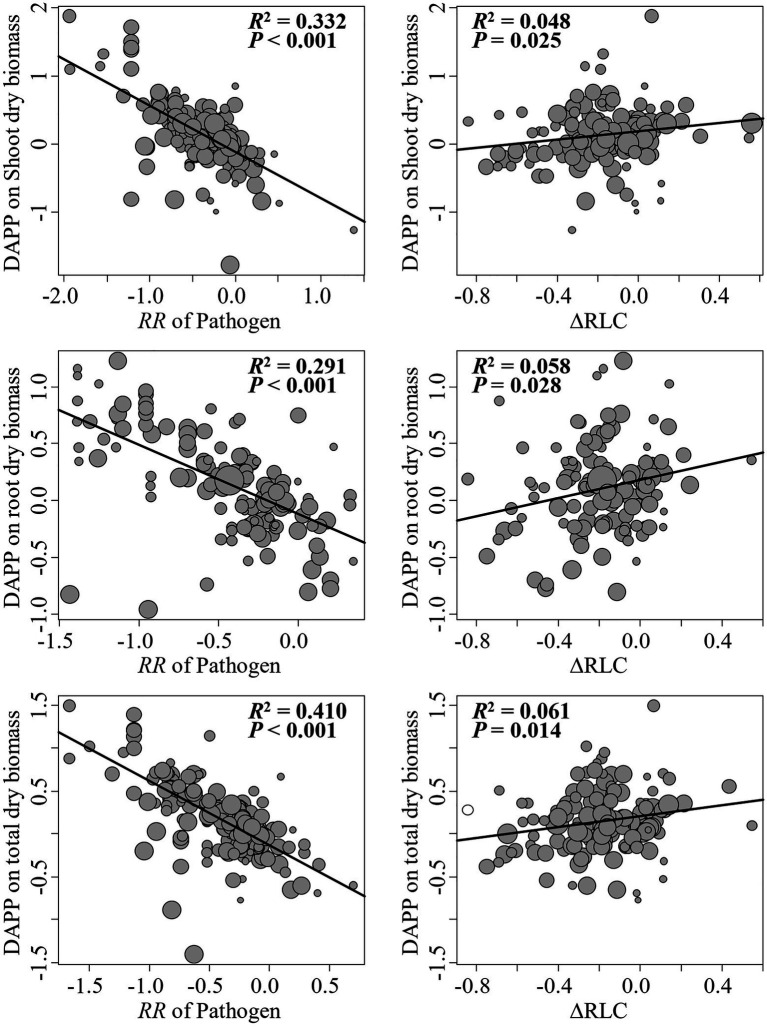
Linear relationships between the effect size difference of AMF on the plant dry biomass under plant pathogenic microbes and no-pathogenic conditions (DAPP) with ∆RLC and effect of plant pathogenic on plant biomass. Significance test for the linear relationship was based on a mixed-effects model with a REML method and values of *p*≤0.05 were significant. The point size is proportional to the weight of each observation in model. N means the number of observations.

The model selection showed that the effect of pathogenic microbe on plant dry biomass was the most important moderator to determine the DAPP (Figure 4; Table 1), which was all negatively correlated with DAPP for shoot, root, and total dry biomass (Table 1; Figure 5). The ∆RLC was the sub-important moderator with a positive effect in influencing the DAPP of all the types of biomass (Figures 4, 5; Table 1). These results revealed that the most important factor to determining DAPP is harm level of plant pathogenic microbes on plant growth, which was negatively correlated. This directly demonstrated that the worse harm to host plant from pathogenic microbes, the greater role in systemic defense AMF would play. This could be related to defense priming; the increased harm of pathogenic microbes would further stimulate defense mechanisms in host plants using AMF (Gianinazzi-Pearson et al., 1996; Pozo et al., 2009; Campos-Soriano et al., 2012). Additionally, this might potentially make the competition of pathogenic microbes with AMF for fatty acid fiercer under the colonization of AMF, which would also reduce the infection of plant microbes in plants and ostensibly increase the effect of AMF on plant growth (Jiang et al., 2017). These results also showed that the presence of AMF could mitigate harmful effects of plant pathogenic microbes and highlighted that the appropriate regimes to maintain AMF diversity and abundance in arable soil are necessary to maintain the health of crops with regard to plant pathogenic microbes. However, our results were calculated from pot experiments, which are simpler systems than agricultural ecosystems. More studies are needed in the future to investigate the actual dependence of diseased crops on AMF through measuring, for example, RLC, AMF abundance, or biomass. We found a better performance for AMF comparing to the normal condition (without pathogenic microbes), despite the reduction of RLC of AMF caused by plant pathogenic microbes (Figure 2). According to the role of the colonization extent (RLC) determining the nutrient exchanging ratio between AMF and host plant and revealing the host plant dependence level on AMF (Treseder, 2013), the lower the reduction in RLC (or even increase) by plant pathogenic microbes, the higher the performance of AMF with pathogenic microbes. This explains the positive correlation between ∆RLC and DAPP. Furthermore, this result also demonstrated that the AMF species and/or host plant, which suffered less RLC reduction by plant pathogenic microbes, has a higher pathogen resistance and should be applied to control the pathogenic microbes. While, we did not analyze the potential impacts of other moderators on the DAPP separately in this meta-analysis, even of which would be significant, our model selection method avoided exaggeration the effect of a single moderator as well as overlapping effects among different moderators in the impact on the DAPP (Terrer et al., 2016; Crawford et al., 2019). Thus, our results are likely closer to the true biological factors influencing the DAPP without overrepresentation of falsely “significant” moderators due to methodological artefacts.

**Table 1:** The details of significant importance of moderators for the effect size difference of arbuscular mycorrhizal fungi (AMF) on the plant biomass under plant pathogenic microbes and no-pathogenic conditions.

Moderator			Importance	Estimate	95% CI		Lower	Upper
Shoot dry biomass				
*RR* of pathogen	1.000	−0.717	−0.838	−0.595
∆RLC	1.000	0.499	0.292	0.706
Root dry biomass				
*RR* of pathogen	1.000	−0.757	−0.868	−0.646
∆RLC	1.000	0.366	0.200	0.533
Total dry biomass				
*RR* of pathogen	1.000	−0.717	−0.838	−0.595
∆RLC	1.000	0.499	0.292	0.706

## Conclusion

This intensive meta-analysis of the interaction between AMF and plant pathogenic microbes significantly advances the understanding of plant pathogenic microbes on the functions of AMF plant growth promotion. The dependence of plant growth on AMF was negatively correlated with the harm level of plant pathogenic microbes on plant and positively correlated with the RLC change ratio and had no significant relationships with other biotic factors through a model selection method. All these results help us to understand the beneficial potential of AMF and to find a more efficient AMF species with regard to resistance to plant pathogen and applications in agroecosystems.

## Data Availability Statement

The original contributions presented in the study are included in the article/Supplementary Material, further inquiries can be directed to the corresponding author.

## Author Contributions

MQ designed the study with the help of WW and YT. MQ, WW, YL, and HF analyzed the data. MQ and J-PM wrote the manuscript with extensive discussion with YT and WW. All authors contributed to the article and approved the submitted version.

## Funding

This study was supported by the Second Tibetan Plateau Scientific Expedition and Research Program (STEP), Grant NO. 2019QZKK0301, the Fundamental Research Funds of China West Normal University (19E048 and 18Q046), the National Natural Science Foundation of China (31870579, 31870494, and 31971445), and the Applied Basic Research Program of Sichuan Province (2020YJ0346).

## Conflict of Interest

The authors declare that the research was conducted in the absence of any commercial or financial relationships that could be construed as a potential conflict of interest.

## Publisher’s Note

All claims expressed in this article are solely those of the authors and do not necessarily represent those of their affiliated organizations, or those of the publisher, the editors and the reviewers. Any product that may be evaluated in this article, or claim that may be made by its manufacturer, is not guaranteed or endorsed by the publisher.

## Footnotes

^1^ http://www.cnki.net/

^2^ http://getdata-graph-digitizer.com

^3^ http://soils.usda.gov

^4^ https://invam.wvu.edu/
