# Supplementary figures and images for: Pathogenic Microbes Increase Plant Dependence on Arbuscular Mycorrhizal Fungi: A Meta-Analysis
Source: Front Plant Sci. 2021 Oct 4;12:707118. doi: 10.3389/fpls.2021.707118 (PMC8521030; doi:10.3389/fpls.2021.707118)

**Supplementary Figure 1** PRISMA flow diagram for this study.


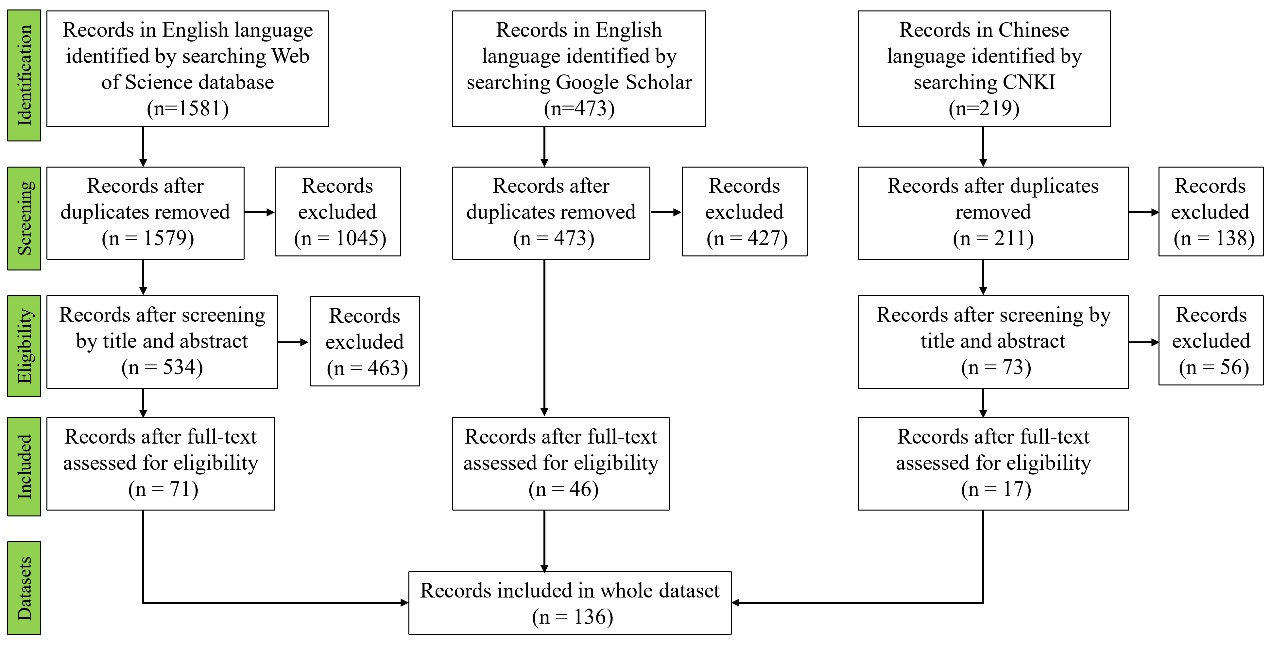

Supplement: Supplementary file 2 [file Data_Sheet_2.docx]
